# Analysis of potential virulence genes and competence to transformation in *Haemophilus influenzae* biotype *aegyptius* associated with Brazilian Purpuric Fever

**DOI:** 10.1590/1678-4685-GMB-2020-0029

**Published:** 2020-12-21

**Authors:** Rafaella Fabiana Carneiro Pereira, Thais Holtz Theizen, Daisy Machado, João Paulo de Oliveira Guarnieri, Gabriel Piccirillo Gomide, Luciana Maria de Hollanda, Marcelo Lancellotti

**Affiliations:** 1 Universidade Estadual de Campinas, Departamento de Bioquímica e Biologia Tecidual, Instituto de Biologia, Campinas, SP, Brazil.; 2 Universidade Estadual de Campinas - UNICAMP, Faculdade de Ciências Farmacêuticas - FCF, Campinas, SP, Brazil.

**Keywords:** Haemophilus influenzae biotype aegyptius, Brazilian Purpuric Fever, virulence, qPCR, competence

## Abstract

Brazilian Purpuric Fever (BPF) is a hemorrhagic pediatric illness caused by *Haemophilus influenzae* biogroup *aegyptius* (Hae), a bacterium that was formerly associated with self-limited purulent conjunctivitis. BPF is assumed to be eradicated. However, the virulence mechanisms inherent to Hae strains associated with BPF is still a mystery and deficient in studies. Here, we aim to analyze the role of the autotransporter genes related to adherence and colonization *las*, *tabA1,* and *hadA* genes through RT-qPCR expression profiling and knockout mutants. Relative quantification by real-time PCR after infection in human cells and infant rat model suggests that *las* was initially downregulated probably duo to immune evasion, *tabA1,* and *hadA* were overexpressed in general, suggesting an active role of TabA1 and HadA1 adhesins in Hae *in vitro* and *in vivo*. Transformation attempts were unsuccessful despite the use of multiple technical approaches and *in silico* analysis revealed that Hae lacks genes related to competence in *Haemophilus*, which could be part of the elucidation of the difficulty of genetically manipulating Hae strains.

## Introduction

*Haemophilus influenzae* biogroup *aegyptius* (Hae) was formerly associated with seasonal epidemics of self-limited purulent conjunctivitis (“pink eye”) until the 1980s, when an emergent clone of Hae was identified as the etiological agent of Brazilian Purpuric Fever (BPF) (Brenner *et al.,* 1988; Harrison *et al.*, 2008). BPF is a fulminant pediatric disease characterized by conjunctivitis, high fever, purpura, and sepsis. BPF major outbreaks arose from 1984 to 1990 in São Paulo, Brazil. Sporadic cases have been registered in Australia, USA (Harrison *et al.*, 2008) and more recently in 2007 in Pará, Brazil (Santana-Porto *et al.*, 2009). BPF is a disease with mandatory reporting in Brazil. The invasive unique phenotype highlights Hae associated with BPF (Hae-BPF) from other *Haemophilus influenzae* strains (Hi). 

Potential virulence factors such as pilus proteins and lipopolysaccharide have been studied *in vitro* with endothelial cells (Quinn *et al.*, 1995; Weyant *et al.*, 1994) and in infant rats (Rubin and St Geme, 1993); however, the origin and virulence mechanisms of Hae-BPF is still unknown. A potential pathogenic determinant in Hae is the *las* gene, a member of the AIDA-I/VirG/PerT family of virulence-associated autotransporters (ATs). Homology and sequence analysis suggest that *las* emerged from Hi *lav* (Davis *et al.*, 2001).

Genome comparative analysis identified that Hae accessory genome has 21 specific Hae-BPF putative genes (Strouts *et al*., 2012; Pereira *et al*., 2019) and a much richer repertoire of AT adhesins in comparison to another available genome of *Haemophilus* genus*.* A total of eight new trimeric autotransporter adhesins (TAAs) was described in Hae (BPF and conjunctivitis isolates) and are present as homologs, termed *tabA* - for the Hae-BPF alleles, or *tahA* - for the Hae non-BPF alleles. TabA1 was characterized as the P145 protein (Strouts *et al.*, 2012), identified in Hae isolates from animals (Rubin and Rizvi, 1991; Rubin, 1995). The *tabA1* and *tahA1* loci are different in the coding sequences, suggesting different functions, and *tabA1*, possess two additional coding sequences, HIBPF*06250* and IS1016 (absent in non-BPF strains) (Strouts *et al.*, 2012). The formerly described as the *Haemophilus* capsulation locus-associated insertion sequence IS1016 has been related with unusual and invasive virulence of non-typeable Hi (NTHI) isolates.

HadA is a TAA unique to BPF associated strains and belongs to the ‘oligomeric coiled-coil adhesin’ (Oca) family found in virulent bacterial isolates. According to Serruto *et al.* (2009), *hadA* gene is missing in Hi Rd KW20 and non -BPF Hae F1947, but these strains present the sequences flanking this gene. Because the GC composition of *hadA* is significantly different from the rest of the Hi genomes (Serruto *et al.*, 2009) and the F3031 genome (Strouts *et al.*, 2012), it is believed *hadA* has been acquired by horizontal transfer (HT) (Serruto *et al.*, 2009).

Since HT is a crucial element in the evolution and emergence of bacterial pathogens, a possible explanation for the clonal emergence of Hae-BPF is the acquisition of one or more virulence genes by HT (Bartlett, 2006, Diep *et al.*, 2006). Hi is a bacterium naturally competent to transformation (Mell *et al.*, 2011, Sinha *et al.*, 2012). Sinha *et al.* (2012) identified the Sxy-dependent cyclic AMP receptor protein (CRP-S) regulon required for natural transformation in Hi. The genetic manipulation in Hae could be an important tool to elucidate the role of virulence genes in Hae-BPF. However, it was only once registered as successful in the literature (Segada and Lesse, 1997); several attempts were made to obtain mutants strains but all failed (Li *et al.*, 2003, Serruto *et al.*, 2009).

 Here, we describe for the first time the expression of three potential virulence-associated AT genes of Hae-BPF in human cells and in the infant rat model. We also tried to elucidate the puzzling enigma of Hae competence to transformation.

## Material and Methods

### Bacterial growth and DNA extraction

Growth conditions and DNA extraction protocol followed the methods described previously (Varela *et al.,* 2014; Cury *et al.,* 2014). Bacterial strains used in this work are described in Table 1. 

**Table 1 -:** Bacterial strains used in this work.

Strain	Alias	Bacteria	GenBank acession no.
F3031	254	Hae-BPF	FQ670178
F3033	258	Hae-BPF	NA
F3030	219	Hae-BPF	LNKQ00000000
F3039	321	Hae-BPF	LNKN00000000
F3042	284	Hae-BPF	LNKO00000000
F3283	406	Hae-BPF	LNKP00000000
F1949	Karina	Hae-BPF	LNKS00000000
F3028	329	Hae-BPF	CP043771
F3037	167	Hae-BPF	CP043772
F3043	55	Hae	CP043811
F3052	232	Hae	CP043810
F3047	73	Hae	FQ670204
KC1018	-	Hae	LNKR00000000
ATCC11116	-	Hae	AFBC00000000
RdKW20	-	Hi type d	L42023
β-lac	Hib-βlac	Hi type b	NA
JM109	-	Competent *E. coli*	-

### *In vitro* infection assays 

*In vitro* infection assays were based on methods described by Pereira *et al.* (2011). Briefly, A549 (human pulmonary epithelial carcinoma) and Hec1b (human endometrium adenocarcinoma) cells were grown in RPMI-1640 medium (Cultilab, Campinas, Brazil) supplemented with 10 % of fetal bovine serum (Gibco, USA). Cells suspensions were seeded in 12-well tissue culture plates and incubated until confluence of cell monolayers. A total of 50 µl of the bacterial suspensions with approximately 1.10^7^ CFU of F3031, F3033, KC1018, and ATCC11116 strains were inoculated in). After an incubation time of 6 hours at 37 °C with 5 % CO_2_, adherent bacteria were recovered in chocolate agar, grown as described previously and used for RT-qPCR analysis. All assays were performed with technical triplicates.

### Animal model infection assays

Litters of Sprague-Dawley rats (*Rattus norvegicus*) were acquired from “Centro Multidisciplinar para Investigação Biológica na Área de Ciência em Animais de Laboratório - CEMIB/UNICAMP” (http://www.cemib.unicamp.br). They were bred in a specific-pathogen-free facility at IB-UNICAMP and were 8 to 10 days old. The animal model and protocol were based on previous studies with Hae (Rubin and Carlone, 1989; Rubin and St Geme, 1993). Briefly, a single dose of 1.10^5^ CFU of bacteria was given intraperitoneally and animal infection was performed in groups as described in Table 2. After a period of 24 and 48 hours, animals were anesthetized by ketamine/xylasin use (50/5 mg/kg) before euthanasia, and blood was obtained by heart puncture. Bacteria were recovered in chocolate agar, grown as described previously, and used for further analysis.

**Table 2 -:** Recovery of animal-passaged strains from infection assays.

Strain	No. animals with positive blood culture/ total	
	24 hours	48 hours
F3031	2/5	0/5
F3033	2/5	2/5
KC1018	0/5	0/5
ATCC11116	0/3	-

The protocol for animal practice was approved in accordance with relevant guidelines and regulations: “Comissão de Ética e Uso de Animais - CEUA - UNICAMP” Comission of Ethics in animal use protocol number: 3197-1 (http://www.ib.unicamp.br/comissoes/ceua)”. 

### RNA extraction

Bacterial isolates from *in vitro* and *in vivo* assays were grown as previously described. Extraction of total RNA was in Trizol Reagent^®^ protocol. RNA concentration and quality were determined by NanoDrop (NanoDrop^®^ 2000 - Thermo Scientific).

### Primer design and validation

Gene sequences were retrieved from F303 genome: *alaS* (alanyl-tRNA synthetase), *era* (ERA-binding protein), *gmk* (guanylate kinase), *gyrA* (subunit A of DNA gyrase), *map* (methionine aminopeptidase), *primase*, *recA* (recombinase A), *recF* (Replication and DNA repair protein RecF), *rho* (Rho transcription termination factor), *rpoA* (alpha subunit of RNA polymerase), *rpoC* (beta subunit of RNA polymerase) and *rpoD* (sigma of RNA polymerase), *las*, *tabA1*, and *hadA*. Primers for qPCR and transformation assays were designed using Primer3Plus software (available at https://primer3plus.com/) and the sequences are listed in Table S1. qPCR primers were designed with the following criteria: total length ranging from 20 to 25 bp, melting temperature of 60 °C, amplicon length ranging from 70 to 150 bp, average GC content of 50 %. All the primers pairs were evaluated with conventional PCR with genomic DNA to check specificity. *tabA* qPCR primers target a conserved region shared between the homologous genes *tabA1* in Hae-BPF and *tahA1* in non-BPF Hae strains previously described (Strouts *et al.,* 2012).

### RNA reverse transcription and real-time PCR

cDNA synthesis and qPCR (RT-qPCR) assays were carried out using the StepOnePlus™ Real-Time PCR System (Applied Biosystems). Reactions were performed with the SuperScript^®^ III Platinum^®^ (Invitrogen) with SYBR Green Fast (Applied Biosystems). Reactions contained 50 ng of total RNA and 600 nM of each primer. PCR conditions were 50 °C for 15 min followed by 95 °C for 2 min, 45 cycles of 95 °C for 15 s and 60 °C for 1min. Primer pair specificity was evaluated by melting curve analysis and gel electrophoresis. Assays were performed with technical triplicates.

### Evaluation of candidate reference genes in Hae

The expression of candidate reference genes was evaluated on three distinct experimental conditions: 1. Bacteria recovered from *in vitro* infection in A549 and Hec1b cells; 2. Bacteria recovered from *in vivo* infection; 3. Bacterial stock samples (no infection passage). The following strains were used: F3031_A, F3031_S, F3033, F3033_S, ATCC11116, β-lac, and β-lac_H. The suffixes _A, _H, _S, and _S48 correspond to samples isolated from A549, Hec1b, and animal infection for 24 and 48 hours, respectively; samples with no suffix correspond to the stock samples (e.g., F3031_S is the F3031 isolated sample from *in vivo* 24 hours infection, and F3031 is the stock sample). The stability of candidate reference genes was performed with the geNorm algorithm (Vandesompele *et al.,* 2002). An expression stability coefficient for candidate genes was obtained and genes with the lower coefficient were considered stable and used for RT-qPCR assays.

### Relative quantification of potential virulence genes in Hae

The expression of *las*, *tabA1*/*tahA1,* and *hadA* genes were evaluated by RT-qPCR on three distinct experimental conditions: 1. Bacteria recovered from *in vitro* infection in A549 and Hec1b cells; 2. Bacteria recovered from *in vivo* infection; 3. Bacterial stock samples (reference group). The following groups were used: 1. F3031: F3031, F3031_A, F3031_H, F3031_S; 2. F3033: F3033, F3033_A, F3033_S, F3033_S48; 3. KC1018: KC1018, KC1018_A, KC1018_H; 4. ATCC1116: ATCC1116, ATCC1116_A, ATCC1116_H. The relative quantification of each gene was calculated using the 2^-ΔΔCt^ with *gyrA*, *rpoC,* and *rpoD* used as reference genes and stock samples used as reference (control) for normalization. 

### Statistical analysis

For statistical analysis, the relative expression of each gene was compared between group samples using One-way analysis of variance (ANOVA), with the Tukey post hoc test. A p-value of 0.05 indicated a statistically significant difference. 

### Construction of mutant knockout vectors of Hae virulence genes

Recombinant DNA protocols such as cloning plasmids, PCR amplifications, restriction digestion, insertion of resistance cassettes and transformation in *Escherichia coli* JM109 were performed as described previously (Hollanda *et al.,* 2011). Detailed information regarding recombinant protocols are available upon request. Briefly, specific regions of *las, tabA1*, and *hadA* were amplified with primers listed in Table S1. The purified PCR product were cloned into pGEMT Easy System II (Invitrogen), and the construction was digested on specific sites allowing the insertion of *ermAM* cassette harboring a gene encoding erythromycin resistance. Positive clones were checked by PCR analysis using an oligonucleotide specific to the target gene and another specific to the *ermAM* cassette.

### Transformation assays in Hae

Hae-BPF strains F3031, F3033, and F1946 growth and transformation experiments were performed with four different approaches based on the protocols with the graphene oxide nanoparticle mesoporous silica SBA15 as previously described (Varela *et al.* 2014), and chemical treatment with calcium chloride (CaCl_2_) followed by a heat shock (Dagert and Ehrlich, 1979): 1.Treatment with SBA15; 2. Chemical and thermal treatment; 3. Chemical and thermal treatment with SBA15; 4. No treatment.

Bacterial cells were incubated with the constructed vectors harboring a disrupted *las*, *tabA1* or *hadA* gene and the SBA nanoparticles at a final concentration of 30 ng for 30 minutes. Subsequently, 1 mL of supplemented BHI broth supplemented with NAD/ hemin 2/10 μg/mL was added and the cells were incubated at 37 °C with 5 % CO_2_ for 4 hours. Cells were then transferred to BHI agar supplemented with NAD/ hemin 2/10 μg/mL and 7 μg/mL of erythromycin and incubated for 48 hours under the same described conditions. All transformation assays were performed in three replicates at least and negative controls were made to ensure the viability and absence of contamination. 

### Comparative transformation in Hae and Hi

Transformation capacity of Hae F3031, F3033, KC1018, ATCCC11116 and established competent strains of Hi (Rd KW20 and β-lac) was compared using the protocol and the pLAN78 *las::ermAM* vector which contains the ATCC11116 *las* gene fused to the *ermAM* resistance cassette (Cury *et al.*, 2014). 

### In silico evaluation of CRP-S genes in Hae

Sequences of the CRP-S regulon genes (*pilA, pilB, pilC, pilD, comA, comB, comE, comF, comE, comO, comP, comEI, pilF2, rec2, ligA, HI0659, HI0660, HI1631, comM, RadC, and ssb*) were obtained from Rd KW20. Sequences were used for a BLASTn search against a custom database with the genomes of strains listed in Table 1. 

## Results

### *In vitro* and *in vivo* assays 

F3031, F3033, KC1018, and ATCC1116 isolates were successfully recovered from A459 and Hec1b infection assays. Animal-passaged isolation is described in Table 2; only Hae associated with BPF were recovered from the animal model. 

### Expression of candidate reference and potential virulence genes in Hae

Twelve genes were evaluated as possible candidates for Hae reference gene for RT-qPCR assays. We obtained an expression stability coefficient for each gene across three different experimental contexts using geNorm ranging from 1.7881 to 4.3479. The genes with the lowest stability coefficient were considered the most stables and were selected: *gyrA* (1.7881), *rpoC* (1.803) and *rpoD* (1.9115) (see Table S2 for all expression stability coefficients). 

**Figure 1.**
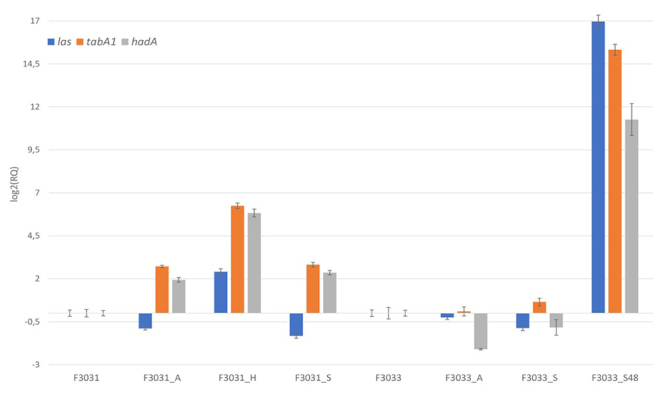
Relative expression of *las*, *tabA1,* and *hadA* genes for Hae-BPF F3031 and F3033 using *gyrA*, *rpoC* and *rpoD* as reference genes. Relative quantification (RQ) data were normalized with control samples (bacteria not recovered from infection assays). Bars represent the error associated with the reported RQ value for each target.

**Figure 2.**
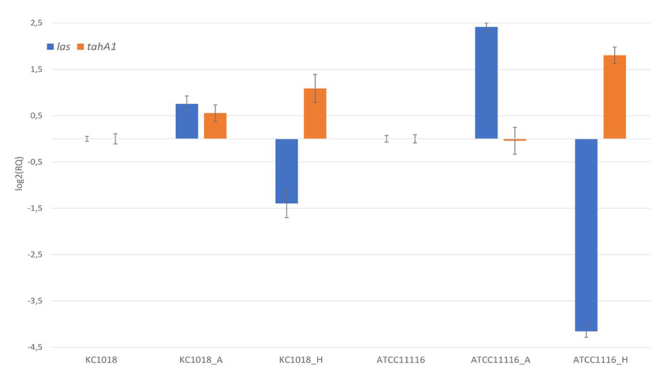
Relative expression of *las* and *tahA1* genes or Hae KC1018 and ATCC11116 using *gyrA*, *rpoC* and *rpoD* as reference genes. Relative quantification (RQ) data were normalized with control samples (bacteria not recovered from infection assays). Bars represent the error associated with the reported RQ value for each target.

We compared the expression of virulence genes of the original strain (stock) with strains recovered from *in vitro* and *in vivo* infection. The relative quantification of *las*, *tabA1*/*tahA1*, and *hadA* genes are presented in Figures 1 and 2 (RQ values are available in Table S3). Hae-BPF cellular and animal-passaged strains showed differentiated expression of the *las* and *tabA1* genes compared to the control samples (p <0.001), except *tabA1* and *las* in F3033_A. The animal animal-passaged strains with different times of infection showed different profiles in *las* expression: the 24 hours samples (F3031_S and F3033_S) showed a significant decrease in *las* expression whereas the F3033_S48 overexpressed this gene (16.9-fold). It is possible that *las* has a potential negative regulation during initial contact with the host, having a more expressive role in the evolution of the disease. In the non-BPF KC1018_H and ATCC11116_H strains, a reduced expression of *las* was observed (p <0.001). 

Expression of *tabA1* in both strains isolated from the animal model (F3031_S and F3033_S48) and F3031 isolated from cells was significantly higher when compared to control stock sample (p <0.001). Increased expression of this gene in F3031 could be related to Hae virulence and survival in the host, since TabA1 is believed to be the P145 protein (Strouts *et al.*, 2012). However, the expression of *tabA1* in F3033 was similar in control and infection samples. In the conjunctivitis samples (KC1018 and ATCC11116), *tahA1* gene was also overexpressed (p <0.001) except in ATCCC11116_A, suggesting a possible role in virulence in non-BPF samples.

Expression of the specific Hae-BPF adhesin/invasin *hadA* were evaluated in F3031 and F3033. F3031_A, H, and S showed an increased expression (p <0.001). Although the F3033_A and S isolates presented not significant changes in *hadA* expression, F3033_S48 showed a higher *hadA* expression (128152-fold change) (p <0.001). The HadA role in the adherence and invasion in human host cells had already been observed (Serruto *et al.,* 2009). 

### Transformation capacity in Hae strains

Since Hae capacity to transform is a controversial subject, new methodologies were used with the addition of silica nanotubes and chemical and thermal treatment. However, we could not obtain a knockout Hae strain for *las*, *tabA1*, or *hadA* gene. The transcriptional fusion vector pLAN78 was used to evaluate the transformation capacity in Hae. pLAN78 was already successfully transferred to *N. meningitidis* and Hi strains (Cury *et al.,* 2014). Hae strains were again not competent to transformation whereas 19 and 4 CFU of Hi RdKW20 and Hi β-lac, respectively, were observed. All negative controls grown, ensuring that the transformation conditions did not compromised the bacterial viability.

BLASTn search results of the twenty-six CRP-S regulon genes in Hae genomes are shown in Table 3. We observed that Hae strains do not have all the genes related to the competence of Hi previously identified by Sinha *et al.* (2012). Three regulon genes, *HI0659*, *HI0660*, and *HI1631*, are absent in all Hae-BPF strains analyzed. Conjunctivitis strains lack the *HI1631* gene.

**Table 3 -:** Analysis of the CRP-S regulon in Hae strains.

Strain	HI0659	HI0660	HI1631
F3031	-	-	-
F3030	-	-	-
F3039	-	-	-
F3042	-	-	-
F3283	-	-	-
F1949			
F3028	-	-	-
F3037	-	-	-
F3043	+	+	-
F3052	+	+	-
F3047	+	+	-
KC1018	+	+	-
ATCC11116	+	+	-
RdKW20	+	+	+

+: presence; -: absence

## Discussion

In animal model experiments, Hae-BPF strains were recovered after 24 and 48 hours of infection while conjunctivitis Hae strains failed to cause bacteremia in infant rats. Rubin and Carlone (1989) had similar data in which Hae associated with BPF caused more expressive bacteremia when compared to non-BPF strains. In a total of 50 infant rats injected with non-BPF strains, only one animal showed bacteremia (Carlone *et al.*, 1989). Those findings suggest that Hae-BPF strains are, not only more virulent but present specific factors involved in immune evasion in infant rats and human hosts. 

 Few genes have been related to Hae unique virulence among which are the ATs *las*, *tabA1*, and *hadA*, whose expression was evaluated in strains after i*n vitro* an *in vivo* infection. Overall results imply that in cellular and animal infection models, *las* is initially downregulated in Hae-BPF. Our data showed that *las* was overexpressed in the Hae_BPF F3033 in the latter stages of the infection. In a previous study, our group showed that the transfer of *las* in NTHi strains caused a higher production of the tumor necrose factor alpha - TNF-α and interleukin 10 - IL10 in Hec1b versus the wildtype strains (Cury *et al.*, 2014). The *las* gene in Hae (*lav* in non-BPF *H. influenzae*) is evolutionarily related to the AT *autB* prone to phase variation in *N. meningitidis* (Davis *et al.*, 2001)*.* Arenas *et al.* (2016) reported that *autB* is switched off in most of *N. meningitidis* isolates probably related to immune evasion. According to their findings, AutB synthesis promotes biofilm formation and may facilitate colonization of host tissues. The Las function in Hae is still unknown. However, our findings indicate a similar role to the meningococcal *autB* and that phase variation could be a major player in the *las* expression in the host.

The specific Hae-BPF genes *tabA1* and *hadA* gene appear to play an active function in infant rats and cell infection. TabA1 has been recognized as the 145-kDa P145 protein, a conserved phase-variable surface protein (Strouts *et al.*, 2012). Although the expression of P145 did not contribute to a pathogenic behavior in the animal model (Rubin, 1995), this AT seems to be important in Hae survival in the host environment, since specific anti-P145 antibody showed bactericidal and protective activity against Hae-BPF in infant rats (Rubin and Rizvi*.*, 1991). Serruto *et al.* (2009) over-expressed HadA in non-pathogenic *E. coli* and observed bacterial aggregation, microcolony formation and invasion in Chang (human conjunctival) and HUVEC (human umbilical vein endothelial) cells. Nevertheless, antibodies against this adhesin may inhibit Hae adhesion in Chang cells. The *hadA* gene is unique to Hae associated with BPF strains, characterized as a determinant of Hae virulence (Serruto *et al.*, 2009; Strouts *et al.*, 2012), although how this adhesin interacts with the host and with other virulence factors is still unknown. 

Here we attempt to genetically manipulate Hae strains to determinate how the knockout of *las*, *tabA1* and *hadA* would affect Hae virulence. After unsuccessful attempts, we hypothesized that, or the mutants were inviable (i.e. the three genes would be essentials to Hae growth and survival), or Hae was unable to transform. We performed a comparative transformation assay with Hae with a vector with a non-deleterious effect to investigate the first hypothesis. Over again, Hae strains associated or not with BPF were not competent to transformation. The unsuccess of genetic manipulation of Hae was already reported (Serruto *et al.*, 2009; Li *et al.*, 2003). Through sequence analysis of CPR-S regulon genes, we discovered that Hae-BPF strains do not harbor the *HI1631*, *HI0659*, and *HI0660* genes. We observed that *HI1631* gene is missing in all Hae strains analyzed - BPF or not. There is no described function and homologs for *HI1631*, a putative gene that is believed to encode a cytoplasmic product related in DNA uptake or transformation (Davis *et al.*, 2001) whose expression is competence induced (Redfield *et al.*, 2005). The difficulty of genetically manipulating Hae remains a mystery, Hae further studies on how the deletion of *HI1631* or other genes could affect competence in Hae are in progress. 

We, the authors, believe that more investigations surrounding the unique virulence of Hae associated with BPF are needed and should be encouraged. Transcriptomic - RNA-seq or array, and proteomic profiling would be a major next step in the characterization of important virulence determinants in Hae-BPF. Heterologous expression of others potential virulence factors besides HadA would be an interesting approach to determine the specific contributions to Hae virulence *in vitro* and *in vivo*.

## Supplementary material

The following online material is available for this article:

Table S1 - Oligonucleotides used in this work.

Table S2 - Stability coefficient (M-value) for candidate genes.

Table S3 - RQ values for *las*. *tabA1*/*tahA1*, and *hadA*.
