# Supplementary material for: Analysis of potential virulence genes and competence to transformation in Haemophilus influenzae biotype aegyptius associated with Brazilian Purpuric Fever
Source: Genet Mol Biol. 2020 Dec 21;44(1):e20200029. doi: 10.1590/1678-4685-GMB-2020-0029 (PMC7816109; doi:10.1590/1678-4685-GMB-2020-0029)
Supplement: Table S1 - [file 1415-4757-GMB-44-1-e20200029-s1.pdf]

Genetics and Molecular Biology

https://doi.org/10.1590/1678-4685-GMB-2020-0029

Supplementary Material to "Analysis of potential virulence genes and competence to transformation in *Haemophilus influenzae* biotype *aegyptius* associated with Brazilian Purpuric Fever"

Table S1 - Oligonucleotides used in this work.

Primer	Sequence 5'-3'	Amplicon lenght (pb)	Application	Reference
***alaS* Forward**	GCACCTTACGCATCAGACAAC	137	Reference gene - qPCR	This work
***alaS* Reverse**	TAACGGTCGCCATCTTCTTC		Reference gene - qPCR	This work
***era* Forward**	CGGTGACGGGTAGTTTGTG	114	Reference gene - qPCR	This work
***era* Reverse**	CGGCTTTATTGCTATCGTAGGTC		Reference gene - qPCR	This work
***gmk* Forward**	TCAATCGCAGGTAAAGAGGTTC	142	Reference gene - qPCR	This work
***gmk* Reverse**	CACGCCCAGGTGAAGTTG		Reference gene - qPCR	This work
***gyrA* Forward**	AAGCAGAGGATGGTATCGTGAG	94	Reference gene - qPCR	This work
***gyrA* Reverse**	GTGGCGGTAAAGGCAAATC		Reference gene - qPCR	This work
***map* Forward**	CTTCCGATGTGCTTGTGATG	147	Reference gene - qPCR	This work
***map* Reverse**	AGGTCGCCTTTGGGAAAC		Reference gene - qPCR	This work
***primase* Forward**	TTGGCTAACTGTGAAGGATGG	144	Reference gene - qPCR	This work
***primase* Reverse**	CGCCCCTTTATTGATGATTTG		Reference gene - qPCR	This work
***recA* Forward**	GGTAATCCTGAAACCACCACAG	72	Reference gene - qPCR	This work
***recA* Reverse**	ACGGCGAATATCTAAGCGAAC		Reference gene - qPCR	This work
***recF* Forward**	TTACGACGAACCGCACTTTAC	94	Reference gene - qPCR	This work
***recF* Reverse**	GGTGTTGCCTTGACGTAGTTTC		Reference gene - qPCR	This work
***rho* Forward**	AAAACAGCACGCCAAAAGTG	82	Reference gene - qPCR	This work
***rho* Reverse**	ACGAAGGAAACCAAAACCATC		Reference gene - qPCR	This work
***rpoA* Forward**	AGCACAACCTGGCATTGAAG	72	Reference gene - qPCR	This work
***rpoA* Reverse**	GAGCGTGGTTTTGGTCATACTC		Reference gene - qPCR	This work
***rpoC* Forward**	GCGGCTAAGAAAATGGTTGAG	114	Reference gene - qPCR	This work
***rpoC* Reverse**	ACCCAAACGGTGAAGTGTTG		Reference gene - qPCR	This work
***rpoD* Forward**	TGCTGTGGCAGAATCAAGAG	85	Reference gene - qPCR	This work
***rpoD* Reverse**	ACCTATTGGCGATGACGATG		Reference gene - qPCR	This work
***las* Forward**	CTTGGTGGCGATGTTTTCTC	82	*las* -qPCR	This work
***las* Reverse**	TGCGTTGTTCTGCTTGTCC			This work

Primer	Sequence 5’-3’	Amplicon lenght (pb)	Application	Reference
tabA1 Forward	TTCCCAAGCCACCAATCAC	85	*tabA1/tahA1* -qPCR	This work
tabA1 Reverse	GCAGCAAATGGCGATAGAAC			This work
hadA Forward	AGCTTGAGCAGCAAGACCAC	94	*hadA* -qPCR	This work
hadA Reverse	GGCAATCGCAACTCACACTC			This work
lasΔ Forward	CAGGCTGTATGACGTCTCCATC	250	Knockout mutant	This work
lasΔ Reverse	GAACAGACTGGAGATTACTGGC		Knockout mutant	This work
tabAΔ Forward	GGTTGTTTCAGAATTAACGAAAGGTG	729	Knockout mutant	This work
tabAΔ Reverse	CAGTTTACGGTGCTATTATTACCAATAG		Knockout mutant	This work
hadAΔ Forward	GCACAAGTCAAAAAAGATGAACTTAGTGAG	470	Knockout mutant	This work
hadAΔ Reverse	GCAAGACCACGTTTAAGATCTTTATTAAGG		Knockout mutant	This work
**ermAM** ***Forward***	AAGCTTGCCGTCTGAATGGGACCTCTTTACTTCTTGG	-	Knockout mutant	Cury *et al*., 2014
**ermAM** ***Reverse***	GCAAACTTAAGAGTGTGTTGATAG		Knockout mutant	Cury *et al*., 2014
