# Supplementary material for: Analysis of potential virulence genes and competence to transformation in Haemophilus influenzae biotype aegyptius associated with Brazilian Purpuric Fever
Source: Genet Mol Biol. 2020 Dec 21;44(1):e20200029. doi: 10.1590/1678-4685-GMB-2020-0029 (PMC7816109; doi:10.1590/1678-4685-GMB-2020-0029)
Supplement: Table S2 - [file 1415-4757-GMB-44-1-e20200029-s2.pdf]

Genetics and Molecular Biology

https://doi.org/10.1590/1678-4685-GMB-2020-0029

Supplementary Material to "Analysis of potential virulence genes and competence to transformation in *Haemophilus influenzae* biotype *aegyptius* associated with Brazilian Purpuric Fever"

Table S2 - Stability coefficient (M-value) for candidate genes.

**Gene**	**M-value**
gyrA	1.7881
rpoD	1.803
rpoC	1.9115
recA	1.9781
alaS	1.9963
map	2.1418
era	2.2083
primase	2.2241
rpoA	2.8559
gmk	4.2245
recF	4.3479
