# Supplementary material for: Analysis of potential virulence genes and competence to transformation in Haemophilus influenzae biotype aegyptius associated with Brazilian Purpuric Fever
Source: Genet Mol Biol. 2020 Dec 21;44(1):e20200029. doi: 10.1590/1678-4685-GMB-2020-0029 (PMC7816109; doi:10.1590/1678-4685-GMB-2020-0029)
Supplement: Table S3 - [file 1415-4757-GMB-44-1-e20200029-s3.pdf]

Genetics and Molecular Biology

https://doi.org/10.1590/1678-4685-GMB-2020-0029

Supplementary Material to “Analysis of potential virulence genes and competence to transformation in *Haemophilus influenzae* biotype *aegyptius* associated with Brazilian Purpuric Fever”

Table S3 - RQ values for *las*. *tabA1*/*tahA1*, and *hadA*.

Strain	***las***			***tabA1/tahA1***			***hadA***		
	RQ	RQ min	RQ max	RQ	RQ min	RQ max	RQ	RQ min	RQ max
**F3031**	1.000	0.879	1.138	1.000	0.858	1.165	1.000	0.900	1.111
**F3031_A**	0.539	0.508	0.571	6.579	6.276	6.898	3.831	3.486	4.211
**F3031_H**	5.319	4.729	5.984	75.888	67.879	84.842	56.673	48.383	66.383
**F3031_S**	0.400	0.363	0.441	7.075	6.437	7.776	5.126	4.680	5.614
**F3033**	1.000	0.875	1.142	1.000	0.791	1.265	1.000	0.888	1.126
**F3033_A**	0.840	0.775	0.909	1.072	0.893	1.287	0.234	0.227	0.240
**F3033_S**	0.548	0.494	0.609	1.571	1.347	1.833	0.562	0.412	0.768
**F3033_S48**	128152.314	98973.136	165934.072	41087.635	32963.701	51213.720	2460.723	1294.609	4677.210
**KC1018**	1.000	0.963	1.039	1.000	0.927	1.079	-	-	-
**KC1018_A**	1.686	1.496	1.901	1.470	1.301	1.662	-	-	-
**KC1018_H**	0.380	0.308	0.469	2.125	1.720	2.626	-	-	-
**ATCC11116**	1.000	0.951	1.052	1.000	0.940	1.064	-	-	-
**ATCC11116_A**	5.337	5.036	5.656	0.972	0.795	1.189	-	-	-
**ATCC1116_H**	0.056	0.051	0.062	3.492	3.088	3.949	-	-	-
